# Effect of Side-Chain Functional Groups in the Immunogenicity of Bacterial Surface Glycans

**DOI:** 10.3390/molecules28207112

**Published:** 2023-10-16

**Authors:** Guangzong Tian, Chunjun Qin, Jing Hu, Xiaopeng Zou, Jian Yin

**Affiliations:** 1 Key Laboratory of Carbohydrate Chemistry and Biotechnology, Ministry of Education, School of Biotechnology, Jiangnan University, Wuxi 214122, China; guangzong.tian@jiangnan.edu.cn (G.T.); chunjun.qin@jiangnan.edu.cn (C.Q.); xiaopeng.zou@jiangnan.edu.cn (X.Z.); 2 Wuxi School of Medicine, Jiangnan University, Wuxi 214122, China; hujing@jiangnan.edu.cn; 3 School of Life Sciences and Health Engineering, Jiangnan University, Wuxi 214122, China

**Keywords:** bacterial surface glycans, functional groups, carbohydrate antigens, biological activities, immunogenicity

## Abstract

Glycans on the surface of bacteria have diverse and essential biological functions and have widely been employed for treating various bacterial infectious diseases. Furthermore, these glycans comprise various functional groups, such as *O*-, *N*-, and carboxyl-modified, which significantly increase the diversity of glycan structures. These functional groups are not only crucial for glycans’ structural identity but are also essential for their biological functions. Therefore, a clear understanding of the biological functions of these modified groups in corresponding bacterial glycans is crucial for their medical applications. Thus far, the activities of functional groups in some biomedical active carbohydrates have been elucidated. It has been shown that some functional groups are key constituents of biologically active bacterial glycans, while others are actually not essential and may even mask the functions of the glycans. This paper reviews the structures of naturally occurring side-chain functional groups in glycans located on the bacterial surface and their roles in immunological responses.

## 1. Introduction

Carbohydrates are the crucial constituent on the surface of many bacterial species, mainly present as capsular polysaccharides (CPSs), glycoproteins, and glycolipids (like lipopolysaccharide, LPS) [1]. These molecules have unique characteristics attributed to specific bacteria and different bacterial serotypes (STs) [2]. Moreover, these molecules play a fundamental immunomodulation role in the host after a pathogen attack and have been widely used in the immune response to protect against pathogenic bacterial infectious diseases [3]. With the progress of immunological evaluation and structural identification of bacterial surface polysaccharides (PSs), various carbohydrate-based vaccines have been developed against infectious diseases [4]. Currently, these carbohydrate vaccines require a fundamental understanding of the immune epitopes of glycan antigens. To produce a robust antibody response against bacterial surface glycans (BSGs), the identification of key epitopes is essential for developing carbohydrate-based vaccines [5]. The epitope characteristics of BSGs are suggested to be substantially associated with the frameshifts, length, terminal sugars, sequence, and side-chain constituents [2]. It is well-known that bacterial PSs often include various non-carbohydrate constituents, including phosphate, acetyl, pyruvate ketal, and amino acids, which increase the structural diversity of BSGs [6,7,8]. Although the biomedical activities of most functional groups in BSGs have not yet been identified, they are considered crucial immunological determinants, constituting an essential part of the immunodominant epitopes. Previous studies have shown that some functional groups in BSGs are essential immunological determinants [5,6,9], while others may mask crucial epitopes from the immune system, thus inhibiting the antibody response and facilitating immune evasion [6,10,11]. Therefore, it is urgent to clarify the biomedical importance of functional groups in more BSGs, which can provide strong guidance for their medical application. The primary reason for the lack of research on the activities of BSG functional groups is the difficulty of obtaining well-defined sugar chains with and without functional groups. Only *O*-acetyl-modified bacterial glycans have been widely studied, as they can be easily removed under alkaline conditions [6]. With the advancements in structural identification and modern synthetic methods, it is easier to acquire well-defined bacterial glycans with or without these non-carbohydrate constituents, providing a means to investigate the biomedical role of these functional groups [5,9,12]. Moreover, the development of analytical techniques, for instance, nuclear magnetic resonance (NMR) spectroscopy and X-ray crystallography, has significantly improved the discovery and identification of non-carbohydrate functional groups and allows the exploration of structure–activity relationships. This review aims to elucidate the presence of side-chain functional groups in BSGs and their currently identified functional association with immune response.

## 2. Naturally Occurring Side-Chain Functional Groups in the Glycans on the Bacterial Surface

Multiple functional groups in BSGs can be linked to sugars in different ways, mainly as *O*-modified, *N*-modified, and carboxy-modified substituents by ester, acetal, ether, and amidic linkages. Identification of the structures and types of these functional groups will allow a better understanding of their biomedical activity.

### 2.1. Bacterial Glycans’ O-Modified Side-Chain Functional Groups

The *O-acetyl* moieties are frequently observed and have been determined as targets for many PS antigens [6,13] (Table 1). On the other hand, various rare *O*-linked acyl groups have been found in some BSGs; for instance, in *V. anguillarum* O-antigen*,* an *O*-linked propanoyl group has been identified [14] (Table 1). *O*-Methylation is also a frequent modification, and parent polymers’ structural and compositional profile revealed that the *O*-methylation is either partial, stoichiometric, or, in some cases, confined to the non-reducing terminus unit [15]. Ethers with (*S*)- and (*R*)-lactic acids, generating 1-carboxyethyl derivatives were observed in O-antigens from multiple *enterobacteria,* such as 4-*O*-[(*R*)-1-carboxyethyl]-D-glucose from *Shigella dysenteriae* [16], while 2-*N*Ac-3-*O*-[(*S*)-1-carboxyethyl]-2-deoxy-D-glucose has been determined as an O-PS component of *Proteus penneri* [17]. In the structure of the O-antigen of *Providencia alcalifaciens*, stereoisomeric 2,4-dihydroxypentanoic acids were found to be linked to different monosaccharides via ether linkages [18]. Cyclic (*R*)- or (*S*)-pyruvate ketals are common in BSGs, e.g., CPS and LPS. Pyruvate groups are usually present as 4,6-*O*-ketals which modify the 4-OH and 6-OH of various monosaccharides [19]. 3,4-*O*-Pyruvate ketal isomeric forms have been found on 3-OH and 4-OH of both D-galactose and L-rhamnose in some BSGs [20]. However, 2,3-*O*-pyruvate ketal-containing sugar residues have been identified only in a few cases; for example, a 2,3-*O*-pyruvate ketal-α-D-galactose was identified in the CPS of *Streptococcus pneumoniae* ST4 [21]. Phosphoric esters are frequently observed in BSGs, mainly interlinking monosaccharides in the PS chain [22,23]. Conversely, they may also attach as substitutes or add an amino alcohol or alcohol to the main chain. Glycerol, 2-aminoethanol, and ribitol are the most frequent phosphate-linked non-sugar parts. There are also some uncommon compounds, such as choline [24] in *Morganella morganii* O-antigens and arabinitol in *H. alvei* 1191 [25].

### 2.2. Bacterial Glycans’ N-Modified Side-Chain Functional Groups

The structural diversity provided by various amino sugars in BSGs, such as CPS, glycoconjugate, LPS, and other exopolysaccharides, is further increased after encountering diverse *N*-acyl substituents (Table 2). Identification of numerous amino sugar structures indicates that amino function is mostly linked with various acyl group substituents and is rarely free-form. The 2-acetamido-4-amino-D-fucose was first observed in the O-antigen of *S. sonnei* as a key residue and always occurs with the free amino group at the *C-*4 position [26]. The amino sugars’ amino group is usually acetylated and formylated; however, acetimidoyl has also been identified in various bacterial glycans [27]. Moreover, amino acids are one of the most important substituents of amino sugars. They contribute to the PS charge and may promote bacterial PS antigens’ immunospecificity [28]. Several *N*-linked amino acids have been discovered, including D- and L-alanine in *E. coli* O161 [29] and *Proteus penneri* 25 [30], and D- and L-aspartic acids in *Treponema medium* ATCC 700293 [31] and *Proteus* spp. [32], glycine in *S. dysenteriae* D7 [15], L-serine in *E. coli* O114 [33], L-threonine in *Pseudoalteromonas agarivorans* KMM 232 [34], and L-allothreonine in *V. cholerae* O43 [35]. Moreover, some 5-oxoproline derivatives have been revealed as amino sugars’ *N*-acyl substituents [36,37,38]. Besides the *N*-linked amino acid derivatives of amino sugars, bacteria utilize different fatty acids to activate the amino sugars for relevant amido group formations (Table 2). Among them, the most abundant are (*R*)- and (*S*)-3-hydroxybutanoic acids that exist in various BSGs [39,40], while 3,5-dihydroxyhexanoic acid was found in *Flavobacterium psychrophilum* O-antigen [41], and 2,3-dihydroxypropionic acid was found in *Pragia fontium* 97U124 [42]. *N*-linked dicarboxylic acids have also been observed, such as malonic and L-malic in *P. mirabilis* [43] and *Pseudoalteromonas rubra* [44], respectively. In addition to the *N-acyl* derivatives, the less common *N*-linked substituent methyl group was also identified in bacterial glycans; for instance, the 2,4-diamino-L-fucose residue that exists in the terminal of *Bordetella pertussis* LPS contains a methyl moiety as a 4-amine functional group [45].

### 2.3. Bacterial Glycans’ Carboxyl-Linked Side-Chain Functional Groups

Various BSGs contain glycuronic acid residues in which the carboxyl groups are linked to the amino group of amino compounds by forming amides [50] (Table 3). The difference in carboxyl-linked substitutes significantly improves the structural variety of the natural carbohydrates. In the simplest examples, these are primary amides-CONH_2_, such as the 2-acetamido-2-deoxy-galacturonamide residue in *P. aeruginosa* O6 O-antigen [51]. The 2-aminopropane-1,3-diol occurs as an amide with the carboxyl group of uronic acids in *S. boydii* O8 O-antigen [50]. The other known amide linkages are formed with the amino groups of various amino acids, including L-alanine in *H. influenzae* type d [52], L-lysine in *P. mirabilis* O27 [53], L-serine in *P. mirabilis* O28 [54], L-threonine in *R. sphaeroides* ATCC 17023 [55], D-allothreonine in *H. alvei* 1206 [23], L-ornithine in *T. medium* ATCC 700293 [31], glycine in *E. coli* O91 [56], and L-glutamic acid in *Klebsiella* K82 CPS [57]. The *N*^ε^-[(*S*)-1-carboxyethyl]-L-lysine, a derivative of L-lysine, has been identified in *P. rustigianii* O14 O-antigen [58].

## 3. The Biological Activity of Bacterial Surface Glycans’ Functional Groups

Recently, many researchers have investigated the biological activities of some functional groups in BSGs. Currently, there are two strategies for studying the activity of modified functional groups: (1) demodification of natural PSs (such as the removal of *O*-acetyl groups under alkaline conditions) to compare the biological activity of PS antigens with or without functional groups; (2) chemical synthesis of oligosaccharide fragments and their derivatives to investigate the contribution of functional groups to the biological activity of PS antigens. Some research results have shown that some functional groups in bacterial glycans are essential immunological determinants and indispensable, while others mask crucial epitopes from the immune system. These biological activities are described below.

### 3.1. Functional Groups in Bacterial Glycans as Essential Determinants for Immunogenicity

**Figure 1:**
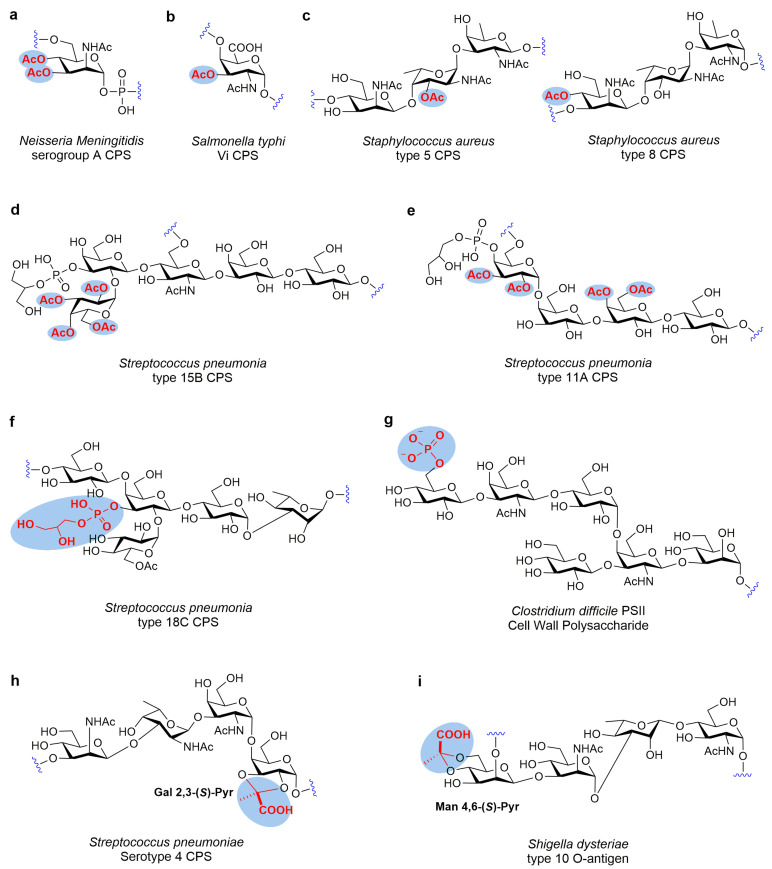
Structures of bacterial surface glycans containing functional groups as essential determinants of immunogenicity. (**a**–**e**) The *O*-acetyl as key constituents of immunogenicity in bacterial glycans. (**f**,**g**) The *O*-phosphoric esters as key constituents of immunogenicity in bacterial glycans. (**h**,**i**) The *O*-pyruvate ketal phosphoric esters as key constituents of immunogenicity in bacterial glycans.

The CPS of *Neisseria meningitidis* serogroup A (sero-A) comprises repeating units of α-1,6-linked *N*-acetyl mannosamine phosphate linked by phosphodiester linkages with partial *O*-acetylation at 3-OH or 4-OH (Figure 1a). A study has shown that the CPS of wild-type meningococcal sero-A comprises 60–70% *O*-acetylated Man*p*NAc residues, primarily at *O*-3 and partly at *O*-4 [59]. Berry et al. [60] utilized human sera and mouse immunization to reveal the significance of *O*-acetyl groups for *Neisseria meningitidis* sero-A CPS immunogenicity. Most post-immunization antibodies interacting with sero-A CPSs were specific for epitopes comprising *O*-acetyl moieties and affected by the de-*O*-acetylation. Immunogenicity studies in mice indicated that the removal of the *O*-acetyl group from the conjugate vaccine substantially reduced its immunogenicity, which is at least 32- and 4-fold lower than the *O*-acetylated CPS–conjugate vaccine and native CPS, respectively. While de-*O*-acetylated CPS was poorly immunogenic, some epitopes without the *O*-acetyl groups elicited weak protective immune responses and induced some bactericidal antibodies. These data suggested that the *O*-acetyl moieties of meningococcal sero-A CPS are essential for CPS immunogenicity. The investigation by Gudlavalleti et al. [59] revealed that for the protection of meningococci from physiological human sera killing, *O*-acetylation is not required; however, this is contradictory to a study of an *O*-acetylation-deficient mutant which confirms the importance of *O*-acetylation in sero-A PS immunogenicity. The study again validates the significance of *O*-acetylation in sero-A PS immunogenicity [59,61,62]. Recently, Adamob et al. [63] clearly elucidated the antigenic epitope of *Neisseria meningitidis* serogroup A through a multidisciplinary approach including inhibition enzyme-linked immunosorbent assay, saturation transfer difference NMR (STD-NMR) spectroscopy, surface plasmon resonance, and X-ray crystallography. A trisaccharide motif with 3-*O*-acetyl at the upstream residue was demonstrated to be the antigenic epitope, and the *O*-acetylated moieties play a crucial role in the binding of trisaccharide with antibodies. Therefore, *O*-acetylation is an important parameter for *Neisseria meningitidis* serogroup A CPS vaccine development and production.

*Salmonella typhi* Vi CPS comprises variably *O*-acetylated (60–90%) repeating α-1,4-linked *N*-acetyl galacturonic acid units at *C*-3 (Figure 1b). Previous immunochemical studies have shown that *O*-acetyl moiety is crucially associated with the immunogenicity of the Vi PS antigen. The Vi CPS immunogenicity was markedly linked with the extent of its *O*-acetylation. Complete de-*O*-acetylation of Vi CPS antigen will eliminate its immunogenicity, but partial de-*O*-acetylation could mildly enhance the immunogenicity. It is noteworthy that although the completely de-*O*-acetylated Vi loses immunogenicity, it still possesses antigenicity and could react with human anti-Vi sera [64,65]. The Courtauld–Koltun space-filling model of a pentamer could explain the dominant activity of the *O*-acetyls in the immunologic behavior characteristic of Vi. This model indicated that most of the Vi surface comprises bulky nonpolar 3-*O*-acetyls as protruding rows on both sides [66]. Bolgiano et al. have explained the experimental phenomena of why antibody binding to the partially de-*O*-acetylated Vi is slightly higher than that of the fully *O*-acetylated form by using molecular dynamics simulations [67]. The results demonstrated that the dynamic behavior and conformation of the Vi CPS were changed after de-*O*-acetylation, from a rigid helix into a more flexible coil, and the hidden epitopes exposed. Thus, the partially de-*O*-acetylated Vi CPSs are more easily recognized by Vi CPS antiserum. Types 5 and 8 of *Staphylococcus aureus* CPSs contain repeating trisaccharide unit of L- and D-Fuc*p*NAc and Man*p*NAcA residues (Figure 1c). The difference between the two CPSs is the site of *O*-acetylation and intra-sugar stereochemical glycosidic linkages. For types 8 and 5 CPSs, the *O*-acetylation is observed at 4-OH of Man*p*NAcA and 3-OH of L-Fuc*p*NAc, respectively [68]. Scully et al. [69] suggested that *O*-acetylation is important for CPS cross-reacting material 197 (CRM_197_) conjugates for efficient killing responses to *S. aureus*, proved by opsonophagocytic killing in vitro assay and protection in the murine pyelonephritis in vivo model. Based on the above results, the authors speculated the failure of the *S. aureus* CPS conjugate vaccines as, although this vaccine primarily mediated robust antibody responses, no marked protection was identified in a clinical phase III trial [70]. One reason could be the manufacturing inconsistency because of alterations in utilized contract facilities; however, the specific effect of these alterations was not elucidated. Furthermore, the reason could be the non-sufficiently sensitive analyses employed to elucidate vaccine quality and critical quality variables, e.g., *O*-acetylation was difficult to identify. Therefore, the only solution is to obtain sufficient materials in a phase III study for direct comparison with the recent preclinical evidence [71].

The structure of the *Streptococcus pneumonia* ST15B CPS is composed of a pentasaccharide repeating unit with *O*-acetylation at 2-OH, 3-OH, 4-OH, and 6-OH of the terminal galactose residue (Figure 1d) [72]. In the work of Rajam et al. [73], the role of *O*-acetylation in type 15B CPS was investigated. The data indicated that the primary functional ST15B CPS epitope is notably linked with the *O*-acetylation of the terminal galactose residue, and removing this *O*-acetyl group inhibits antibody functions. The absence of cross-reactive antibodies in post-vaccinated sera to ST15C with CPS similar to the ST15B but naturally de-*O*-acetylated further validated the above results. The authors speculated that the loss of *O*-acetylation alters capsular polymers’ antigenic structure, making them nonreactive to the anti-15B antibodies. *Streptococcus pneumonia* ST11A CPS is a linear heteropolymer consisting of a tetrasaccharide repeating unit with a pendant phosphoglyceride and *O*-acetylation pattern (Figure 1e). There is an average of 2.6 *O*-acetyls per repeating unit in the ST11A CPS. Zartler et al. [74] have examined the recognition process of anti-ST11A serum to ST11A CPS by using an inhibition-type ELISA and flow cytometry. Research showed that the immunoreactivity of ST11A pneumococcal CPS will decrease after de-*O*-acetylation, demonstrating that *O*-acetylation plays a crucial role in the antigenicity of these PSs. Recent studies have shown that *O*-acetylation modification of polysaccharides in serotypes 28F and 28A affects the affinity between *Streptococcus pneumoniae* and antiserum factor 23d, and the reaction between *O*-acetylated CPS and antibodies is weakened, but not completely lost [75]. In addition, studies have shown that *O*-acetylated *Escherichia coli* is more virulent than de-*O*-acetylated *E. coli* [76].

*S. pneumoniae* ST18C CPS comprises repeated pentasaccharides and is markedly branched by glycerol phosphate and D-glucose (Figure 1f) [77]. The loss of the glycerol phosphate group may inhibit the desired immediate ST18C CPS-induced response. The de-phosphorylated fragments of 18C CPS were employed to inhibit sera with 18C PS antibodies to elucidate the function of the glycerol phosphate group in CPS recognition [78]. The data show that no sera were completely suppressed by de-P-Gro, indicating that the glycerol phosphate group is important for recognizing ST18C CPS. Therefore, it is necessary to preserve the glycerol-phosphate to conserve adequate antigenicity of the ST18C CPS.

The hexaglycosyl phosphate constitutes the repeating polyanionic PS unit of the *Clostridium difficile* PSII cell wall [79] (Figure 1g). To elucidate if the single repeating unit attached to the carrier protein can sufficiently produce anti-PSII antibodies in mice and if synthetic oligosaccharide phosphorylation is necessary, Adamo et al. [80] established the repeating units of phosphorylated hexasaccharide PSII and its nonphosphorylated-counterpart-bearing linker at the reducing end and conjugated the carrier protein CRM_197_ as a glycoconjugate vaccine against *C. difficile* [81]. Immunological evaluation of the glycoconjugates in mice revealed that in synthetic glycans, the phosphate group is essential for mimicking the native PSII PS. In mice, the CRM_197_ conjugated native PSII and repeating synthetic phosphorylated hexasaccharide unit demonstrated comparable immunogenic responses [81]. This was the first investigation to highlight that phosphorylation of the terminal residues comprising phosphodiester-linked glycopolymer short-length fragments is a crucial index of their immunogenicity. These data furnish novel perspectives on the design and carbohydrate antigen selection and highlight the importance of phosphodiester linkages in the surface-exposed PSs of various pathogenic bacteria as vaccine candidates.

The *Streptococcus pneumoniae* ST4 CPS comprises the repeated tetrasaccharide unit and has an acid labile *trans*-(*S*)-2,3-*O*-pyruvate [20,82] (Figure 1h). Although *S. pneumoniae* ST4 CPS is an important constituent of Prevnar 13, a commercial vaccine, the role of *trans*-2,3-(*S*)-pyruvate has not been studied. Furthermore, the crucial ST4 repeat unit epitope is still undetermined, and no previous research elucidating the effect of pyruvate modification on immunogenicity exists. This carbohydrate modification is significant for establishing new vaccines, as inferred in the 1970s [83]. Seeberger et al. [5] answered these questions by first synthesizing the repeating unit of ST4 tetrasaccharide, its depyruvated derivative, and deletion sequences for comprehensive immunological research. To elucidate ST4 repeating units’ antigenicity, microarray slides were utilized for printing the synthetic structures via the reducing ends’ aminophenyl linker for selective immobilization by attaching to the activated carboxylic groups. Isolated PSs were printed as controls, including pneumococcal cell wall polysaccharide (CWPS) and ST4 CPS. The acquired glycan microarray was labeled with rabbit anti-ST4 typing polyclonal serum, specifically raised in isolated ST4 CPS immunized animals. Increased native ST4 CPS antibody levels were observed but not those of CWPS or other STs. For synthetic oligosaccharides, distinct interaction was identified exclusively for pyruvalated tetrasaccharides and not for depyruvalated deletion sequences. These results highlight the significance of the *O*-pyruvate in stimulating an immune response against the native PSs and indicate that for generating minimal synthetic carbohydrate vaccines against *S. pneumoniae* ST4, pyruvate modification is crucial.

*Shigella dysenteriae* ST 10 O-antigen contains a repeating unit of a tetrasaccharide [→2)-β-D-Man*p*4,6(*S*)Pyr-(1→3)-α-D-Man*p*NAc-(1→3)-β-L-Rha*p*-(1→4)-α-D-Glc*p*NAc-(1→], in which a (*S*)-4,6-*O*-pyruvyl ketal is located on the β-Man residue (Figure 1i) [84]. To elucidate the immune epitopes of this antigen, its chemical synthesis is essential. Our group [12] designed and generated the non-pyruvylated, and (*R*)- and (*S*)-4,6-*O*-pyruvylated tetrasaccharides and three other fragments related to the *S. dysenteriae* ST10 O-antigen tetrasaccharide. All synthetic oligosaccharide fragments were immobilized on microarray slides and tagged with the anti-serum of *S. dysenteriae*. The specific interaction only with the (*S*)-4,6-*O*-pyruvalated tetrasaccharide was observed, and the results indicated that the (*S*)-4,6-*O*-pyruvyl ketal is a crucial structural characteristic of ST10 O-antigen and is significant for establishing carbohydrate-based vaccines against *S. dysenteriae* ST10.

### 3.2. Functional Groups in Glycans on the Bacterial Surface Are Not Essential for Inducing Functional Antibody Responses

**Figure 2:**
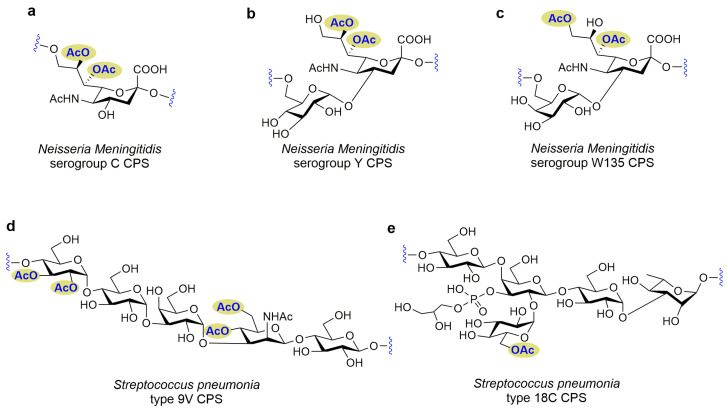
Structures of bacterial glycans containing functional groups are not essential for inducing functional antibody responses. (**a**–**e**) The *O*-acetyl are not essential constituents for immunogenicity in bacterial glycans.

*Neisseria Meningitidis* serogroup C CPS is an α-(2,9)-linked Neu5Ac homopolymer, substantially *O*-acetylated (>90%) at *C*-7 and *C*-8 (Figure 2a). The *O*-acetyl moiety of freshly extracted serogroup C CPS is most frequently present at *C*-8 and less frequently at *C*-7. However, after the serogroup C CPS purification and storage, most *O*-acetyl moieties migrate from *C*-8 to *C*-7 [85]. A previous study has shown that an unconjugated serogroup C CPS vaccine in de-*O*-acetylated form was significantly immunogenic in humans, evidenced by the clinical trials of the 1970s and 1980s, which suggested that *O*-acetylation was not necessary for vaccine immunogenicity [86,87,88]. Therefore, it was suggested that the de-*O*-acetylated form of the serogroup C CPS could substitute the *O*-acetylated vaccine for all age groups [88]. Michon et al. [10] indicated that serogroup C CPS antigens’ immunogenicity highly depended on the extent of *O*-acetylation. Vaccinating completely or partially de-*O*-acetylated serogroup C CPS–tetanus toxoid conjugate in mice caused enhanced serum bactericidal activity (SBA) against the *O*-acetylated serogroup C strain C11. The serogroup C CPS–tetanus toxoid conjugate vaccine was well-tolerated and markedly immunogenic in UK adults, children, and infants. A serological study using competitive inhibition SBA assays suggested that the serogroup C CPS-based protective bacterial epitope is de-*O*-acetylated. Unlike *O*-acetylation in the sero-A CPS, the *C*-7 and *C*-8 *O*-acetylation in meningococcal serogroup C was not directly linked with high immunogenicity. In addition, the *O*-acetyl group in ST C CPS masks the protective epitope and forms less immunogenic epitopes through steric hindrance or altered conformations, thus escaping immune surveillance.

*Neisseria Meningitidis* serogroup Y CPS comprises a disaccharide repeating unit [→6)-α-D-Glc*p*-(1→4)-α-D-Neu*p*5NAc-(2→], which is a heteropolymer with *O*-acetylation at the *C*-7 and *C*-9 positions of Neu5Ac (Figure 2b) [89]. The *O*-acetyl moiety is most frequently observed at the C-7 position on the surface of serogroup Y CPS, and after migration, most of these groups relocate to the *C*-9 position. Fusco et al. [11] investigated the meningococcal Y antigen and indicated the same phenomenon as in group C meningococcal, namely that the *O*-acetyl hides the epitope from the immune system and inhibits antibody response, resulting in immune escape. The de-*O*-acetylated CPS-TT conjugate vaccines are more immunologically competent than the CPS and can elicit more functional antibodies against the protective epitopes.

*Neisseria meningitidis* serogroup W135 CPS is also a heteropolymer comprising repeating α-(2,4)-linked sialic acid disaccharides attached to the *O*-6 position of galactose residues (Figure 2c) [89]. Although most clinical serogroup W135 isolates are not *O*-acetylated, some strains indicate *O*-acetylation at *C*-7 or *C*-9 of the sialic acid residue. In addition, based on the requirements of the WHO Expert Committee on Biological Standardization, the currently licensed W135 CPS–conjugate vaccines have a minimum *O*-acetyl content of 0.3 mmol/g of PS [90]. To determine if *O*-acetylated W135 CPS is essential for optimum immunogenicity, Gudlavalleti et al. [91] indicated that *O*-acetyl groups do not contribute a dominant epitope in raising bactericidal antibodies, consistent with Jin et al.’s investigation [92].

*Streptococcus pneumonia* ST9V CPS, one of the four capsular types (9V, 9N, 9L, and 9A) in Group 9, is an important component of the multivalent pneumococcal vaccines. It comprises a repeating unit of unbranched linear pentasaccharide with *O*-acetyl groups at the *C*-2 and *C*-3 positions of the Glc*p* residue and *C*-4 and *C*-6 positions of the Man*p*NAc residue (Figure 2d) [93]. Mcneely et al. [94] compared the relative contributions of the *O*-acetylated ST9V CPS and de-*O*-acetylated backbone in the immune response of a human and an infant rhesus monkey. Antibodies were detected against the *O*-acetylated ST9V CPS and de-*O*-acetylated backbone. In addition, the opsonophagocytic antisera, the predominant antibody, identified the de-*O*-acetylated ST9V CPS. Therefore, the authors suggested that since no *O*-acetyl groups were recognized, they could not induce antibody responses against ST9V CPS [94]. For the *Streptococcus pneumonia* ST18C CPS with *O*-acetyl moieties at *C*-6 of the Glc*p* residue (Figure 2e) [77], *O*-acetylation was only marginally involved in recognition and was not linked with antigenicity and immunogenicity [78]. Therefore, the de-*O*-acetylated ST18C CPS fragment could be used for conjugate vaccine production.

All Group B *streptococcal* (GBS) capsules express an antigenically unique structure, a terminal α-(2,3)-linked Neu5Ac residue [95], which inhibits complement-induced killing. In several GBS STs (V, VI, III, II, Ib, and Ia), *O*-acetylations were present at the glycerol chain of the Neu5Ac residues [96] and attenuated GBS Sia-mediated neutrophil suppression and virulence [97]. Initially, *O*-acetyl is mostly present at the *C*-7 position but subsequently migrates at the *C*-8 and *C*-9 positions. Although the development of the GBS vaccine primarily involves de-*O*-acetylated CPS, investigating the effect of their formulations on sera activity against *O*-acetylated strains is crucial. A study utilized 20 clinical isolates (V, III, II, Ib, or Ia) of GBS CPS with variable *O*-acetylation (2~40%) to elucidate healthy adult sera immunized with de-*O*-acetylated GBS CPS-TT conjugate vaccines in opsonophagocytosis. It revealed the killing of all strains and >90% opsonophagocytosis, suggesting that de-*O*-acetylated CPS–conjugate vaccines comprise immunogenic epitopes that protect against GBS, independent of *O*-acetylated CPS. Therefore, *O*-acetyl moieties on the GBS CPS are insignificant for GBS glycoconjugate-vaccine-induced functional antibodies [98].

## 4. Conclusions

BSGs often contain an array of side-chain functional groups, usually as *O*-modified, *N*-modified, and carboxyl-modified substituents, such as *O*- or *N*-acetyl, *O*-phosphate and pyruvate, amino acids, etc. Modified functional groups on these sugar rings further enhance bacterial PSs’ structural diversity and biomedical activity. In previous studies, the biological activities of some bacterial glycan functional groups have been revealed, but many non-carbohydrate substituents are still unknown. Therefore, it is important to reveal the biological activity of this modification present in bacterial glycans, which will greatly increase the medical application of biomedical active carbohydrates.

This review summarizes the biological functions of some modification groups in BSGs, some of which are essential determinants of biomedical roles, such as *O*-acetyl in meningococcal sero-A, *Salmonella* typhi Vi, *S. aureus* type 5 and 8, *O*-phosphate in *Clostridium difficile* PSII Cell Wall, 2,3-*O*-pyruvate ketal in *S. pneumoniae* ST 4, and 4,6-*O*-pyruvyl ketal in *S. dysenteriae* ST10, while other modifications may mask their biomedical functions, such as *O*-acetyl in meningococcal serogroups C and Y. The currently available results indicated that most studies are focused on the effect of *O*-acetyl moieties on immune response. Meanwhile, studies on the biological activities of other substituents, such as pyruvate, *O*-phosphate, and *N*-modified or carboxy-modified amino acids, are relatively scarce. The main reason is that the *O*-acetyl moieties can easily be eliminated in alkaline pH, but there is no feasible method for removing all functional groups without causing damage to the parent chain structure. Although the *O*-acetyl content of bacterial glycans is a key quality control factor, it has not yet been employed to monitor the manufacturing of glycoconjugate vaccines.

Currently, a synthetic approach, such as chemical and enzymatic synthesis, is the most feasible method for investigating the biological activities of functional groups in BSGs. In this way, it is easy to acquire well-defined carbohydrates with or without the functional groups, and even the degree of modification can be controlled. The advances in synthetic and modern analytical techniques, such as STD-NMR, immunoassay technology, and X-ray crystallography, are expected to permit better investigation of the biological functions of various BSG functional groups and widen the biomedical application of biomedical active carbohydrates.

## Figures and Tables

**Table 1:** The known *O*-modified functional groups in bacterial PS antigens.

*O*-Modified Groups	Representative Bacterial Polysaccharides	Glycan	References
acetyl	→4)-*β*-D-Glc*p*NAc3RA-(1→4)-*α*-L-Fuc*p*NAm3R-(1→3)-*α*-D-Sug*p*-(1→	*Flavobacterium columnare* ATCC 43622 *O*-antigen	[13]
propanoyl	*β*-L-Qui*p*3NAc-(1-[→4)-*β*-L-Qui*p*3NAc-(1→4)-[*α*-D-Qui*p*NAc3/4R-(1→2)]-*β*-L-Qui*p*3NAc-(1-]→	*Vibrio anguillarum* O-antigen	[14]
methyl	*α*-D-Man*p*3OR-(1-[→3)-*β*-D-Man*p*-(1→2)-*α*-D-Man*p*-(1→2)-*α*-D-Man*p*-(1-]→	*Klebsiella* O5 and *Escherichia coli* O8 O-antigen	[15]
(*S*)-1-carboxyethyl	→3)-*β*-D-Glc*p*NAc6OAc-(1→6)-*β*-D-Glc*p*NAc3OR-(1→3)-*α*-D-Gal*p*-(1→	*Proteus penneri* 62 O-antigen	[17]
2,4-dihydroxypentanoic acid 2-ethers	→4)-β-D-Gal*p*NAc-(1→3)-*β*-D-Gal*p*NAc-(1→3)-[*β*-D-Man*p*4OR-(1→4)]-*α*-D-Gal*p*-(1→	*Providencia alcalifaciens* O31 O-antigen	[18]
4,6-*O*-pyruvate ketal	→3)-*α*-D-Qui*p*NAc-(1→4)-[*α*-D-Gal*p*NAc4,6R-(1→6)]-*α*-D-Gal*p*NAc-(1→4)-*α*-D-Gal*p*NAcA-(1→	*Acinetobacter baumannii* D78 CPS	[19]
3,4-*O*-pyruvate ketal	→3)-*β*-D-Glc*p*NAc-(1→4)-[*β*-D-Gal*p*3,4R-(1→3)]-*β*-D-Gal*p*NAc-(1→4)-*β*-D-Glc*p*NAc-(1→	*P. mirabilis* O24 O-antigen	[20]
2,3-*O*-pyruvate ketal	→3)-*β*-D-Man*p*NAc-(1→3)-*α*-L-Fuc*p*NAc-(1→3)-*α*-D-Gal*p*NAc-(1→4)-*α*-D-Gal*p*2,3R-(1→	*Streptococcus pneumoniae* ST4 CPS	[21]
phosphoric ester	→6)-*α*-D-Glc*p*-(1→2)-*β*-D-Glc*p*-(1→3)-*β*-D-Glc*p*NAc-(1→3)-[*β*-L-Rha*p*-(1→4)]-*α*-D-Glc*p*NAc-(1-PO_3_H→	*E. coli* O152 O-antigen	[23]
glycerol-P- and choline-P-	→4)-[*α*-D-Gal*p*N-(1→3)]-*β*-D-Gal*p*2PCho-(1→3)-*β*-D-Gal*p*NAc6OAc-(1→3)-Gro-1-*P*-(O→	*Morganella morganii* O-antigen	[24]

**Table 2:** The known *N*-modified functional groups in bacterial PS antigens.

*N*-Modified Groups	Representative Bacterial Polysaccharides	Glycan	References
free amino	→4)-*α*-L-Alt*p*NAcA-(1→3)-*β*-D-Fuc*p*NAc4N-(1→	*Shigella sonnei* phase I *O*-antigen	[26]
acetyl	→4)-*α*-D-Gal*p*NR-(1→4)-*β*-D-Glc*p*NR3NRA-D-(1→3)-*α*-D-Fuc*p*NR-(1→3)-*α*-D-Qui*p*NR-(1→	*Pseudomonas aeruginosa* O1 *O*-antigen	[46]
formyl	→4)-*α*-Pse*p*4OAc5NAc7NR-(2→4)-*β*-D-Xyl*p*-(1→3)-*α*-D-Fuc*p*NAc-(1→	*Pseudomonas aeruginosa* O8 *O*-antigen	[46]
acetimidoyl	→4)-*β*-D-Man*p*NAc3NRA-(1→4)-*β*-D-Man*p*NAc3NAcA-(1→3)-*α*-D-Fuc*p*NAc-(1→	*Pseudomonas aeruginosa* O5 *O*-antigen	[46]
D-alanyl	→8)-*α*-Leg*p*5NAc7NR-(2→4)-*β*-D-Glc*p*A-(1→3)-*β*-D-Glc*p*NAc-(1→	*E. coli* O161 *O*-antigen	[29]
L-alanyl	→4)-*β*-D-Glc*p*A-(1→3)-*β*-D-Glc*p*NAc-(1→6)-[*α*-D-Glc*p*A-(1→4)]-*β*-D-Glc*p*NR-(1→	*Proteus penneri* 25 *O*-antigen	[30]
D-aspartyl	→4)-*β*-D-Glc*p*NAc3NAcA-(1→4)-*β*-D-Man*p*NAc3NA(L-ornithine)-(1→3)-*β*-D-Glc*p*NAc-(1→3)-*α*-D-Fuc*p*4NR(1→	*Treponema medium* ATCC 700293 glycoconjugate	[31]
*N*-acetyl-glycyl	→3)-*β*-D-Qui*p*4NR-(1→4)-*α*-D-Gal*p*NAc3OAcAN-(1→4)-*α*-D-Gal*p*NAcA-(1→3)-*α*-D-Glc*p*NAc-(1→	*S. dysenteriae* D7 *O*-antigen	[15]
*N*-acetyl-D-aspartyl	→6)-*α*-D-Glc*p*NAc-(1→4)-*α*-D-Gal*p*A-(1→3)-*α*-D-Glc*p*NAc-(1→3)-*β*-D-Qui*p*4NR-(1→	*Providencia stuartii* O33 *O*-antigen	[47]
*N*-acetyl-L-aspartyl	→3)-*β*-D-Glc*p*NAc-(1→3)-[*β*-D-Qui*p*4NR-(1→4)]-*β*-D-Gal*p*-(1→6)-*β*-D-Glc*p*NAc-(1→3)-*β*-D-Gal*p*-(1→	*Providencia alcalifaciens* O4 *O*-antigen	[32]
*N*-acetyl-L-seryl	→3)-*α*-D-Glc*p*NAc-(1→4)-*β*-D-Qui*p*3NR-(1→3)-*β*-D-Rib*f*-(1→4)-*β*-D-Gal-(1→	*Escherichia coli* O114 *O*-antigen	[33]
*N*-acetyl-L-threonyl	→3)-*α*-D-Fuc*p*NR-(1→3)-[*β*-D-Man*p*NAcA-(1→4)]-*α*-D-Gal*p*NAc-(1→3)-*α*-L-Rha*p*-(1→	*Pseudoalteromonas agarivorans* KMM 232 *O*-antigen	[34]
*N*-acetyl-L-allothreonyl	→3)-*β*-D-Qui*p*4NR-(1→3)-*α*-D-Gal*p*NAcA-(1→4)-*α*-D-Gal*p*NAc-(1→3)-*α*-D-Qui*p*NAc-(1→	*V. cholerae* O43 *O*-antigen	[35]
(2*S*,4*S*)-*N*-[1-carboxyethyl]-alanyl	→4)-[*β*-D-Qui*p*4NR-(1→6)]-*α*-D-Gal*p*NAc-(1→6)-*α*-D-Glc*p*-(1→4)-*β*-D-Glc*p*A-(1→3)-*β*-D-Gal*p*NAc-(1→	*P. alcalifaciens* O35 *O*-antigen	[48]
*N*-[(*S*)-3-hydroxybutyryl]-D-alanyl	→3)-*β*-D-Qui*p*4NR-(1→6)-*α*-D-Glc*p*NAc-(1→3)-*α*-L-Qui*p*NAc-(1→3)-*α*-D-Glc*p*NAc3OAc-(1→	*E. coli* O123 *O*-antigen	[49]
2,4-dihydroxy-3,3,4-trimethylpyroglutamoyl	→3)-*α*-D-Gal*p*NAcAN-(1→4)-*α*-D-Gal*p*NFoA-(1→3)-*α*-D-Qui*p*NAc-(1→3)-*β*-D-Vio*p*NR-(1→	*Vibrio anguillarum* V-123 *O*-antigen	[36]
3-hydroxy-2,3-dimethyl-5-oxoprolyl	→2)-*β*-D-Qui*p*3N**R**-(1→3)-*α*-L-Rha*p*2OAc-(1→3)-*α*-D-Fuc*p*NAc-(1→	*P. shigelloides* O74 *O*-antigen	[37]
(*R*,*R*)-3-hydroxy-3-Methyl-5-oxoprolyl	→3)-*β*-D-Qui*p*NAc4NAc-(1→4)-[*α*-D-Fuc*p*3NR-(1→3)]-*β*-D-Man*p*NAcA-(1→	*Vibrio cholerae* O5 *O*-antigen	[38]
(*R*)-3-hydroxybutyryl	→4)-*β*-D-Glc*p*NAc3NRA-(1→4)-*α*-L-Fuc*p*NAm3OAc-(1→3)-*α*-D-Qui*p*NAc-(1→	*P. shigelloides* O51 *O*-antigen	[39]
(*S*)-3-hydroxybutyryl	→3)-*α*-L-Pne*p*NAc4OAc-(1→4)-*α*-L-Fuc*p*NAc-(1→4)-*α*-L-Fuc*p*NAc-(1→4)-*α*-L-Fuc*p*NAc-(1→3)-*β*-D-Qui*p*NAc4NR(1→	*Plesiomonas shigelloides* O1 *O*-antigen	[40]
(3*S*,5*S*)-3,5-dihydroxyhexanoyl	→4)-*α*-L-Fuc*p*NAc-(1→3)-*α*-D-Qui*p*2NAc4NR-(1→2)-*α*-L-Rha*p*-(1→	*Flavobacterium psychrophilum* 259-93 *O*-antigen	[41]
D-glyceroyl	→3)-*α*-L-Fuc*p*NAc-(1→3)-*α*-L-Fuc*p*NAc-(1→3)-*β*-D-Qui*p*NAc4NR-(→	*Pragia fontium* 97U124 *O*-antigen	[42]
L-maloyl	→4)-*α*-L-Gal*p*NAm3OAcA-(1→3)-*α*-Sug*p*-(1→4)-*β*-D-Glc*p*NAc3NRA-(1→	*Pseudoalteromonas rubra* ATCC 29570T *O*-antigen	[44]
methyl	*α*-D-Glc*p*NAc-(1→4)-*β*-D-Man2NAc3AcA-(1→3)-*β*-L-Fuc*p*2NAc4NR-(1→6)-[*α*-LD-Hep*p*-(1→4)]-*α*-D-Glc*p*NAc-(1→	*Bordetella pertussis* LPS	[45]

**Table 3:** The known carboxyl-linked functional groups in bacterial PS antigens.

Carboxyl-Modified Groups	Representative Bacterial Polysaccharides	Glycan	References
carboxamide	→3)-*α*-L-Rha*p*-(1→4)-*α*-D-Gal*p*NAc3OAcAR-(1→4)-*α*-D-Gal*p*N(formyl)A-(1→3)-*α*-D-Qui*p*NAc-(1→	*P. aeruginosa* O6 O-antigen	[51]
2-aminopropane-1,3-diol	→3)-*β*-D-Glc*p*NAc-(1→2)-*β*-D-Gal*p*3OAc4OAcA6NR-(1→3)-*β*-D-Gal*p*NAc-(1→4)-*β*-D-Glc*p*A-(1→	*Shigella boydii* O8 O-antigen	[50]
L-alanine/L-serine/L-threonine (2:2:1)	→4)-*β*-D-GlcpNAc-(1→3)-*β*-D-Man*p*NAcA6NR-(1→	*Haemophilus influenzae* type d CPS	[52]
R_1_ = L-lysine, R_2_ = L-alanine	→3)-[*β*-D-Glc*p*NAc-(1→4)]-*β*-D-Glc*p*A6NR_1_-(1→3)-*α*-D-Gal*p*A6NR_2_-(1→3)-*β*-D-Glc*p*NAc-(1→	*Proteus mirabilis* O27 O-antigen	[53]
R_1_ = L-serine, R_2_ = L-lysine	→4)-*α*-D-Gal*p*A6NR_2_-(1→4)-*α*-D-Gal*p*-(1→3)-*α*-D-Gal*p*4OAcA6NR_1_-*β*-D-Glc*p*NAc-(1→	*Proteus mirabilis* O28 O-antigen	[54]
L-threonine	→4)-*α*-D-Glc*p*A6NR-(1→4)-*α*-D-Glc*p*A-(1→4)-*α*-D-Glc*p*A-(1→	*Rhodopseudomonas sphaeroides* ATCC 17023 LPS	[55]
D-allothreonine	→4)-*α*-D-Gal*p*A6NR-(1→2)-*α*-L-Rha*p*-(1→2)-*β*-D-Rib*f*-(1→4)-*β*-D-Gal*p*-(1→3)-*β*-D-Gal*p*NAc-(1→	*Hafni. alvei* 1206 O-antigen	[23]
L-ornithine	→4)-*β*-D-Glc*p*NAc3NAcA-(1→4)-*β*-D-Man*p*NAc3NA6NR-(1→3)-*β*-D-Glc*p*NAc-(1→3)-*α*-D-Fuc*p*4NAsp(1→	*Treponema medium* ATCC 700293 glycoconjugate	[31]
glycine	→4)-*α*-D-Qui*p*3NAcyl-(1→4)-*β*-D-Gal*p*-(1→4)-*β*-D-Glc*p*NAc-(1→4)-*β*-D-Glc*p*A6NR-(1→3)-*β*-D-Glc*p*NAc-(1→	*E. coli* O91 O-antigen	[56]
L-glutamic acid	→3)-*β*-D-Glc*p*-(1→3)-[*β*-D-Glc*p*A6NR-(1→4)]-*β*-D-Gal*p*2OAc-(l→3)-*α*-D-Gal*p*-(l→	*Klebsiella* K82 CPS	[57]
*N*^ε^-[(*S*)-1-carboxyethyl]-L-lysine	→4)-*α*-D-Gal*p*NAc-(1→3)-*α*-D-Glc*p*NAc-(1→3)-*α*-D-Gal*p*A6NR-(1→	*Providencia rustigianii* O14 O-antigen	[58]
